# Survival outcomes of hepatocellular carcinoma resection with postoperative complications – a propensity-score-matched analysis

**DOI:** 10.1097/MD.0000000000006430

**Published:** 2017-03-24

**Authors:** Kenneth S.H. Chok, Millies M.Y. Chan, Wing Chiu Dai, Albert C.Y. Chan, Tan To Cheung, Tiffany C.L. Wong, Wong Hoi She, Chung Mau Lo

**Affiliations:** a Department of Surgery, The University of Hong Kong; b Department of Surgery, Queen Mary Hospital; c State Key Laboratory for Liver Research, The University of Hong Kong, Hong Kong, China.

**Keywords:** complications, hepatectomy, hepatocellular carcinoma, liver resection, survival

## Abstract

Curative resection remains the only hope of cure for hepatocellular carcinoma (HCC), but postoperative complications can have a significant impact on long-term survival. However, only scarce data on such impact can be found in the literature.

This retrospective study reviewed the prospectively collected data of patients who underwent primary liver resection for HCC at our hospital during the period from December 1989 to December 2014. Patients with and without postoperative complications were compared. A 1:1 propensity score matching was adopted by matching age, comorbidity, Model of End-stage Liver Disease score, tumor stage, and extent of resection.

Totally 1710 patients were eligible for the study. Four hundred and sixty-one (27.0%) of them developed postoperative complications while 1249 (73.0%) did not. After propensity score matching, 922 patients were compared in a 1:1 ratio (461 with postoperative complications and 461 without). Patients who developed postoperative complications were demographically similar to patients who did not, but had more intraoperative blood loss and transfusion (both *P* < 0.001), longer hospital stay (17 vs 9 days; *P* < 0.001), worse hospital mortality (12.1% vs 0%; *P* < 0.001), and shorter overall survival (*P* < 0.001). On multivariate analysis, factors that might have affected overall survival were cancer stage (HR 1.22, *P* < 0.001), tumor size (HR 1.02, *P* = 0.005), tumor number (HR 1.08, *P* < 0.001), venous invasion (HR 1.38, *P* = 0.003), extent of resection (HR 1.19, *P* = 0.045), intraoperative blood loss (HR 1.11, *P* < 0.001), postoperative complication (HR 1.37, *P* < 0.001), and era effect (HR 1.27, *P* = 0.01).

Patients should be monitored closely after HCC resection. Prompt treatment of postoperative complications may be salvational.

## 1 Introduction

Hepatocellular carcinoma (HCC) is the most common primary liver cancer, with a high prevalence in Asia and an increasing incidence in Western countries.^[1]^ It is the 3rd most common cancer causing deaths in Hong Kong.^[2]^ The resectability rate for HCC is only 20%, and hence its prognosis is generally poor.^[3,4]^

Postoperative complications may have adverse effects on the long-term outcomes of surgeries.^[5,6]^ Studies of colorectal surgery have suggested that postoperative anastomotic leakage increases the likelihood of tumor recurrence and threatens long-term patient survival after colorectal resection.^[5,6]^ High morbidity and high mortality are often seen after liver resection for HCC with background cirrhosis.^[7]^ This study was to determine the impact of postoperative complication on the survival outcomes of curative resection for HCC.

## 2 Methods

This retrospective study reviewed the prospectively collected data of patients who underwent primary liver resection for HCC at our hospital during the period from December 1989 to December 2014. All operations were standardized and were operated by the same team of surgeons. This study did not require specific institutional approval since our institution permits the use of clinical data in retrospective studies provided that no patient can be identified.

### 2.1 Diagnosis and perioperative management

Diagnosis of HCC was based on the typical imaging finding (ie, early arterial enhancement with early portovenous washout) on computed tomography or magnetic resonance imaging and/or a serum α-fetoprotein level >400 ng/mL; results of HBsAg and anti-HCV tests were also taken into account. Adequate hepatic functional reserve and absence of extrahepatic disease were prerequisites for liver resection. Moreover, the tumor had to be anatomically resectable as evaluated by imaging studies. Hepatic function assessment was by Child–Pugh classification^[8]^ or indocyanine green (ICG) clearance test. From 1989 to 1994, the decision for a hepatectomy was based mainly on Child–Pugh classification. Child–Pugh class C was regarded as a contraindication. After the ICG retention safety limit for major hepatectomy was determined in 1995,^[9]^ suitability for hepatectomy was based largely on ICG clearance test result rather than Child–Pugh class. Patients with an ICG retention rate ≤14% at 15 minutes were eligible for major hepatectomy.^[10]^ Our liver resection technique and postoperative management protocol have been described in previous reports.^[3,11]^ Patients were encouraged to mobilize as early as possible after operation. Diagnosis of recurrence was based on the typical imaging finding; percutaneous fine-needle aspiration cytology was also conducted if radiological results were doubtful. Since 2010, dual-tracer positron emission tomography was performed when indefinite recurrences were encountered.^[12]^ A standardized aggressive management protocol as described in a previous report was adopted to treat recurrences.^[13]^

### 2.2 Statistical analysis

Resection of ≥3 liver segments (according to the Couinaud classification) was regarded as a major resection; otherwise it was a minor resection. Hospital death was death occurring during the hospital stay for the primary operation or within 30 days of the operation. Postoperative complication was defined as any deviation from the normal postoperative course with the need for pharmacological, surgical, endoscopic or radiological intervention, and all postoperative complications were graded according to the Clavien–Dindo classification.^[14]^ Pearson chi-squared test was used to compare categorical variables. Student *t* test and the Mann–Whitney *U* test were used to compare continuous variables. The Kaplan–Meier method was used in survival analyses, and the log-rank test was used for survival comparison. *P* < 0.05 denoted statistical significance, and all *P* values were 2-tailed. A 1:1 propensity score matching (PSM) was performed by matching age (*P* = 0.778), comorbidity (*P* = 0.895), Model of End-stage Liver Disease score (*P* = 0.394), tumor stage (according to the International Union Against Cancer tumor-node-metastasis staging system, 7th edition) (*P* = 0.320), and extent of resection (major versus minor) (*P* = 0.830) with the nearest neighbor procedure by the software R (version 3.2.0 [2015-04-16]). Other analyses were performed by the computer software SPSS, version 18.

## 3 Results

A total of 1710 patients were eligible for the study. Four hundred and sixty-one (27.0%) of them developed postoperative complications while 1249 (73.0%) did not. After PSM, 922 patients were compared in a 1:1 ratio (461 with postoperative complications and 461 without). Table 1 is a comparison of preoperative and operative characteristics between patients with and without postoperative complications, and Table 2 is a comparison of pathological data of the 2 groups of patients. The types and incidences of postoperative complications are shown in Table 3. The study period, which spanned 26 years, was divided into 2 halves – period 1 (1989–2001) and period 2 (2002–2014) – which were then compared. Period 2 saw significant improvements in 4 respects, which can be viewed in Table 4.

**Table 1:**
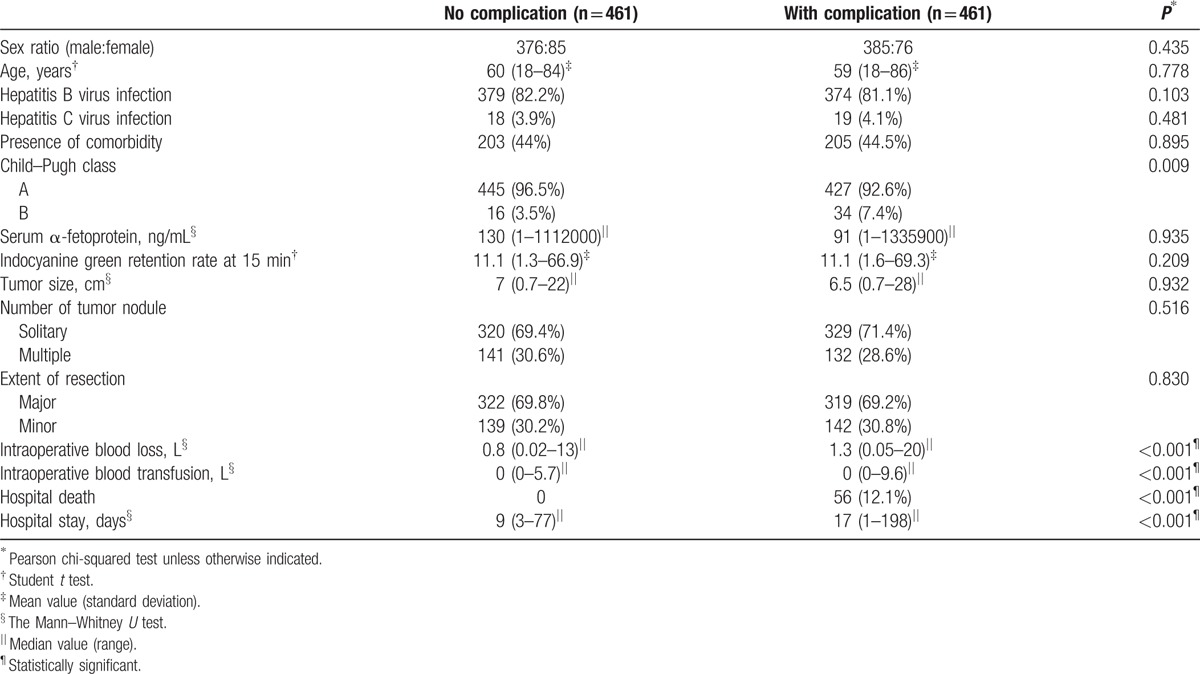
Preoperative and operative characteristics of patients with and without postoperative complications.

**Table 2:**
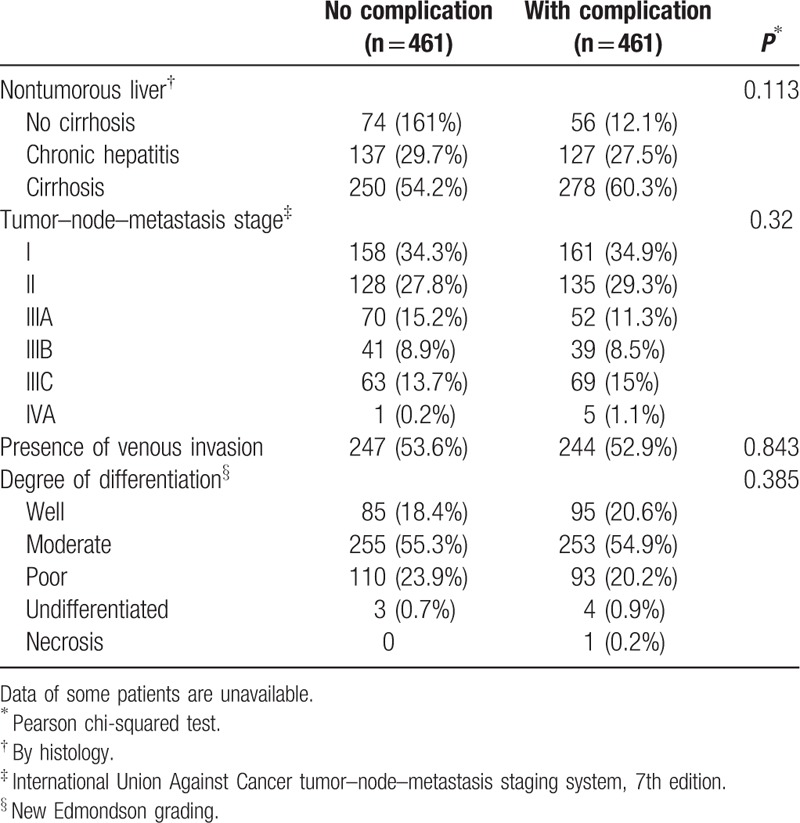
Pathological data of patients with and without postoperative complications.

**Table 3:**
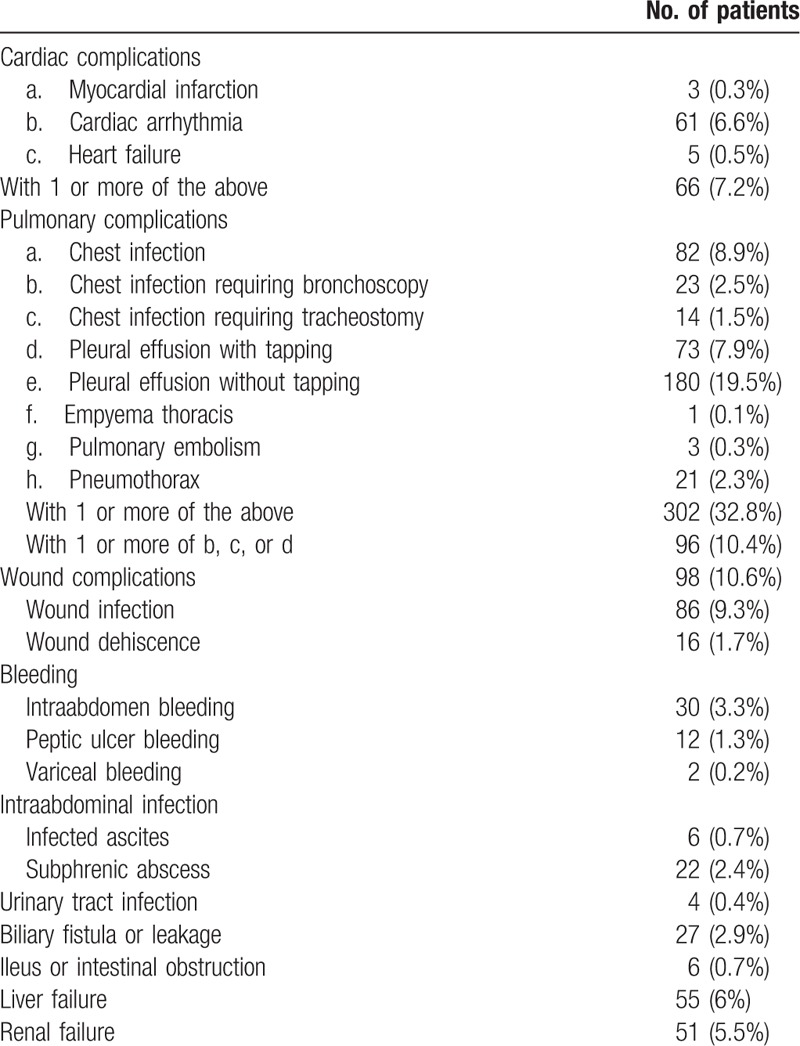
Types and incidences of postoperative complications.

**Table 4:**
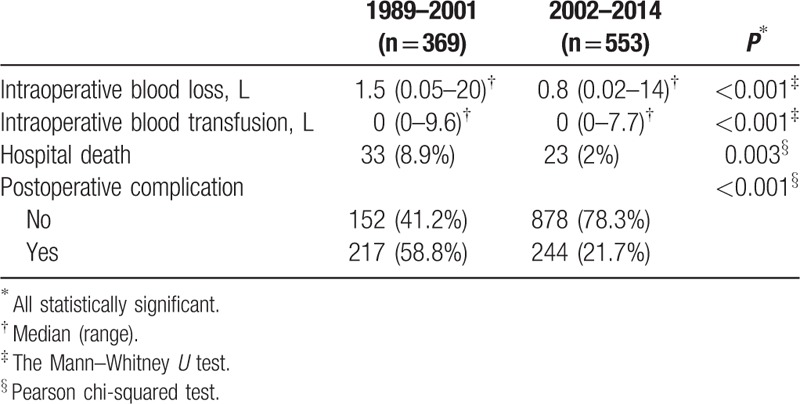
Comparison of short-term outcomes in the 2 periods.

**Figure 1:**
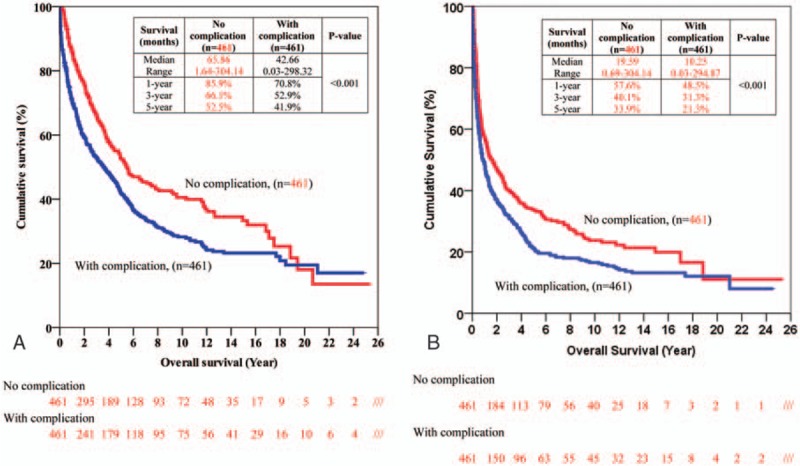
(A) Overall survival of patients with and without postoperative complications. (B) Disease-free survival of patients with and without postoperative complications.

Patients with and without postoperative complications had significantly different overall survival (Fig. 1A) and disease-free survival (Fig. 1B). Table 5 shows the univariate and multivariate analyses of factors that might have affected overall survival. Among the factors was postoperative complication. The impact of different types of postoperative complication on overall survival is shown in Table 6. Overall survival in period 2 was better than that in period 1 (Table 5).

**Table 5:**
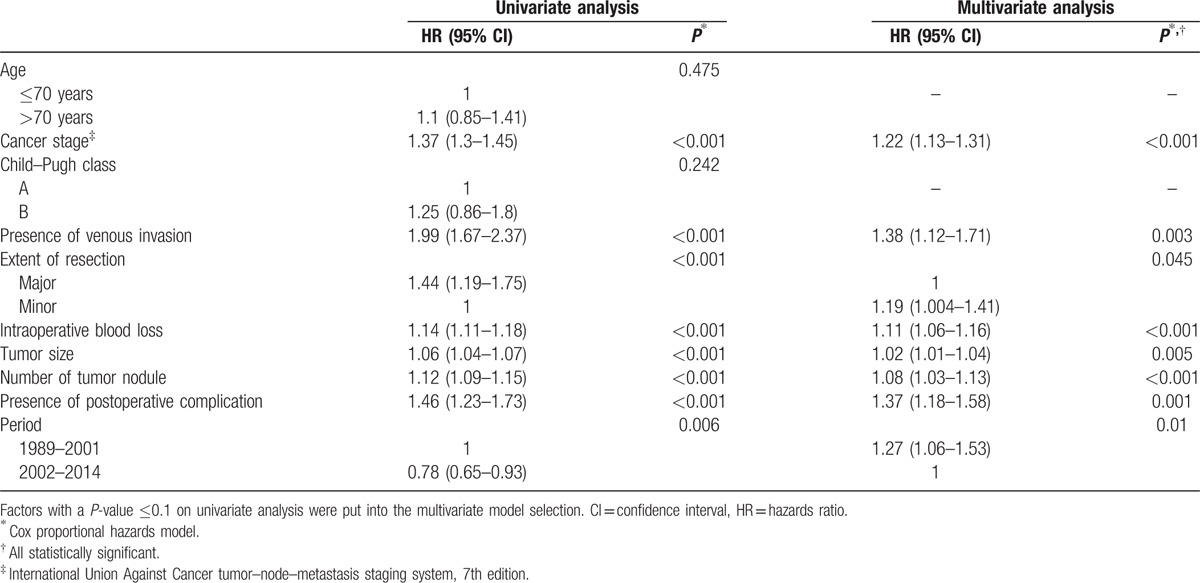
Univariate and multivariate analyses of factors that might have affected overall survival.

**Table 6:**
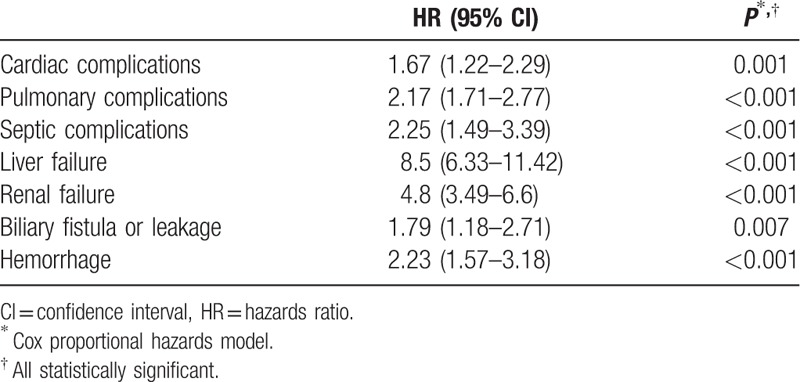
Impact of different types of postoperative complication on overall survival.

## 4 Discussion

With advances in surgical skills and perioperative care, the mortality rate after major hepatectomy has decreased from 58%^[6]^ to <10%.^[15]^ Although primary liver transplantation is another option for curing HCC within criteria, the shortage of grafts limits its applicability. Liver resection is still considered to be the 1st-line treatment, especially for patients with relatively preserved liver function.

Certain factors have been found to affect overall survival after major hepatectomy, which include tumor stage, tumor biology, and postoperation complication. The 1 factor that can be altered by clinicians is postoperative complication. A number of studies have demonstrated the impact of postoperative complication on survival, but the classification of complications differed in them; a couple of them did not use the Clavien–Dindo classification.^[16,17]^ In the study by Kusano et al,^[18]^ which analyzed the postoperative course of 291 patients, the presence of postoperative complication was identified on multivariate analysis as one of the significant factors that affected overall survival. In the study by Okamura et al,^[19]^ this factor was also identified on univariate analysis, though not on multivariate analysis.

The relationship between postoperative complication and survival is a complicated one. Different mechanisms causing such a relationship have been posited. Farid et al^[20]^ saw a survival disadvantage after liver resection for colorectal liver metastasis in patients with complications, and postoperative sepsis was found to be one of the independent factors associated with overall survival and disease-free survival on multivariate analysis. They looked into the septic complications and found that intraabdominal and respiratory infections were associated with poorer long-term outcomes whereas wound infections were not. So far the only kind of postoperative complications whose mechanism is better understood is septic complications. Evidence from studies of other malignancies like colorectal cancer has shown that proinflammatory cytokines such as TNFα and IL-1β increase tumor cell adhesion to endothelial cells in vitro and in vivo by upregulating the expression of cell adhesion molecules.^[21–23]^ Most posited mechanisms are about how infective complications affect oncological outcomes. As for cardiac or renal complications, they may be confounding factors in the prediction of overall survival; further studies are needed before a definitive answer can be given.

Besides careful preoperative assessment and patient selection, intraoperative blood loss should be minimized. The likelihood of postoperative complication seems to correlate with the amounts of intraoperative blood loss and transfusion, as shown in this study as well as previous studies.^[24,25]^ Intraoperative blood loss and postoperative complication have been shown to be associated with overall survival and disease-free survival.^[26]^ Ways of reducing blood loss include maintaining a low central venous pressure and selective use of the Pringle maneuver. Blood transfusion should be avoided as far as possible as it may contribute to an immunocompromised state and affect long-term oncological outcomes.^[27]^ At our center, permissive anemia in asymptomatic patients is allowed and blood transfusion is perioperatively avoided as far as possible. The use of ultrasonic dissector for liver resection has been shown to reduce blood loss and morbidity.^[28]^ On the other hand, a Japanese group showed that the clamp-crush technique produced similar results.^[29]^ Other advanced energy sources may also help to reduce intraoperative blood loss, but proper randomized controlled trials are required to confirm their effectiveness. However, the results of any transection technique are likely affected by the experience and expertise of the handling surgeons and centers.

Among all reported studies of the relationship between postoperative complications and survival outcomes of HCC resection, the present study is the first one to employ PSM. With this method, the impact of selection bias in the estimation of causal effects in observational studies can be reduced. By conditioning on the propensity score, some of the characteristics of a randomized controlled trial can be replicated. A randomized controlled trial, if carried out appropriately, can balance out both measured and unmeasured baseline variables. With PSM, only the distribution of certain selected measured baseline variables between 2 groups in an observational study can be balanced. Therefore, there can still be unbalanced baseline characteristics which are not measured. Nevertheless, since a randomized controlled trial on the topic would be impractical, using PSM is probably the closest method.

This study has limitations other than its retrospective nature. The study spanned 26 years, during which surgical techniques and perioperative care have much improved, and morbidity and mortality have thus significantly reduced, as can be seen in Table 4. This complicated the interpretation of the results. Furthermore, all operations were performed by the same team of surgeons at a single center. Although standardization in patient care and surgical technique can be assured, whether the same outcomes can be replicated elsewhere is unknown.

In conclusion, while there are factors which surgeons have no control of (eg, cancer stage, tumor size and number, and presence of venous invasion), every surgeon should do their best to minimize intraoperative blood loss, and meticulous perioperative care should be given to every patient to prevent postoperative complications, which can significantly affect survival. Should any postoperative complication develops, prompt treatment should be given. Although the exact relationship between postoperative complication and oncological outcome has not been established, there is emerging evidence showing that postoperative morbidity shortens disease-free survival. It is hoped that further research will bring about further reduction in postoperative morbidity and introduce more effective adjuvant treatments for postoperative complications.

## Acknowledgments

The authors thank Mr Henry Tam of Department of Surgery, The University of Hong Kong for performing the statistics in this study.
